# Temperament Characteristics of Children in Residential Care and Perceived Acceptance/Rejection and Style of Discipline Used by Care Workers

**DOI:** 10.3390/bs14121239

**Published:** 2024-12-23

**Authors:** Sabina D. Gaitán, Joanna Fernández-Sánchez, Francisco Javier Fernández-Baena, Agustín Wallace, María D. Salas

**Affiliations:** 1 Departamento de Psicología Evolutiva y de la Educación, Facultad de Psicología y Logopedia, Universidad de Málaga, 29010 Málaga, Spain; joanna.f.s@uma.es (J.F.-S.); m.salas@uma.es (M.D.S.); 2 Departamento de Psicobiología y Metodología de las Ciencias del Comportamiento, Facultad de Psicología y Logopedia, Universidad de Málaga, 29010 Málaga, Spain; awallace@uma.es

**Keywords:** temperament, residential care, acceptance, rejection, style of discipline

## Abstract

The ability to adapt interpersonal interactions to temperamental characteristics is essential for high-quality care. We analyzed how temperamental and self-regulation differences among children in residential care were related to the affective relationships and discipline styles of their caregivers. A total of 144 children aged 9–16 years (42.6% boys) and their caregivers from 22 residential care homes (Spain) participated. The Early Adolescent Temperament Questionnaire-Revised (EATQ-R) was used to assess temperament, the Affect Scale and Rules and Demands Scale was used to assess children’s perceptions of affective relationships and discipline styles among their caregivers, and BRIEF-2 was used to assess children’s self-regulation. Perceived warmth/communication was significantly higher than criticism/rejection and children perceived more inductive than rigid or permissive styles. Temperamental-scale fear was positively related to warmth/communication and an inductive style, and negatively related to criticism/rejection and a rigid style, whereas high-intensity pleasure showed the opposite pattern. In addition, some self-regulation and temperament scales explained 26% of the perception of warmth/communication, while others explained 15% of the variability of the rigid discipline style used by care workers. These results can help care workers to adjust their educational strategies according to the temperamental characteristics of this specific population.

## 1. Introduction

The separation of a child from his or her biological family may be due to several reasons. These include an environment that is inadequate for the child’s correct care and development. In such situations, the administration tries to find protective measures under family care (foster care or adoption). These measures should be adapted to the needs of these children and seek to offer them a safe and stable environment while their biological family’s situation improves. However, there are cases where the characteristics or needs of the children may hinder the implementation of this type of measure. In these cases, an alternative measure is provided: residential care (RC). 

Despite the efforts to guarantee adequate coverage of the needs of children in RC, there are difficulties in ensuring that these needs are met in an optimal manner. These difficulties are inherent both to the profile of the fostered population and to collective protection measures. Hence, studies focusing on the psychological adjustment or development of children in RC repeatedly show the difficulties and problems they face in comparison to the general population [1,2]. However, few studies provide clarifying data on what factors are related to the different difficulties of these children. Both the profile of the children and the characteristics of residential care are essential elements that are not sufficiently reflected in the literature, although they should be analyzed.

### 1.1. Characteristics of Childhood and Adolescence in RC: Experiences of Mistreatment and Adversity and Individual Differences

Firstly, it should be noted that almost all of these young people will have experienced some form of neglect or mistreatment in childhood; therefore, they are at high risk of developing different problems or disorders, i.e., mental health problems [3], behavioral disorders [4], and/or attachment disorders [5]. 

Although most children in residential care have experienced very serious adversity throughout their lives, the level of the impact is not the same for all of them. To understand the variability in the adjustment of children who have experienced adversity, the question should be raised as to why some people are relatively well-adjusted despite the adversity they have experienced, while others are not. Currently, the entire scientific community agrees that people are not simply passive recipients of the experiences we undergo, but that we play an active role and are dynamic builders of our development [6].

Likewise, the developmental temperament theory of Rothbart and colleagues [7] argues that there are biological differences in temperamental traits, namely reactivity and self-regulation. Precisely, such temperamental reactivity refers to the responses (emotional and behavioral) of individuals to changes in the external and internal environment (latency, duration, and intensity). Rothbart’s studies show that the initial responses (from birth) of a child are characterized by physiological and behavioral reactions to sensory stimuli of different intensities or types and are relatively stable over time. According to these authors, in general, temperament determines individual differences that can be observed before the development of other cognitive aspects of personality.

In our field of study (the development of children in RC), individual differences in temperament and, specifically, in reactivity, can play a very important role in psychological development and adjustment. Taking temperament into account would help to partially explain the variability in adaptation and also contribute to defining strengths, vulnerabilities, and opportunities to optimize psychological intervention in this vulnerable and diverse population.

In this sense, the model of Rothbart and colleagues takes into account in their studies about temperament the developmental context and the discipline styles of parents or caregivers [8]. Therefore, the authors emphasize that temperament influences the child’s development through the self-regulation system during a prolonged period in childhood and adolescence. Hence, they stress that throughout this period, there are numerous opportunities for the success or failure of the self-regulation system (first innate regulation in early childhood and then more intentional regulation in childhood and adolescence), and that environmental factors play an important role in facilitating this adaptation (i.e., the context of upbringing and the discipline styles of the caregivers).

### 1.2. Relationships Between Parenting Styles and Children’s Temperament

No studies have been found in the field of RC that establish a connection between fostered children’s temperament and the relationships they have with the care workers and/or their parenting style. However, when studying these interactions in families, studies show that parenting strategies can buffer the effects of temperament on reactivity and self-regulation [9,10]. When children are very young, their reactive tendencies may dominate their behavior, and it is common for them to rely primarily on their caregivers to help them regulate themselves. Thus, sensitive parenting includes the ability to regulate activity levels in response to the child’s behavior; such parental regulation is especially important for children with more reactive temperaments or children with greater negative affectivity. For example, it has been found that children with difficulties in emotional regulation, i.e., lack of control, are more likely to develop externalizing problems when their mothers use more negative parenting styles, characterized by low affection, more control, inflexibility, or intrusive behaviors [11].

The relationship between children’s temperament and their adjustment may be influenced by the parenting styles used by their parents, and these strategies in turn may depend on factors related to the parents themselves or to the temperamental characteristics of the children. This means that the child’s temperament can also influence the choice of certain parenting strategies [12]. Bidirectional studies show that the appearance of problematic behaviors depends on both the individual characteristics of children, parenting styles, and the interaction of the two [11]. It has been found that temperamental characteristics can induce parenting behaviors, indicating child-evoking effects [13,14]; conversely, some parenting styles can also lead to specific temperamental traits [10,15,16].

These bidirectional effects on parent–child relationships have also been investigated through longitudinal studies that analyze the child-evoking effects in middle childhood (6–11 years) [15,17]. Such studies have examined parenting behaviors (e.g., acceptance, discipline, and engagement), showing that the child-evoking effects of low-self-regulating children with a high negative affect predicted more rigid parenting styles approximately four years later. On the other hand, high scores in rigid parenting strategies also predicted lower self-regulation and higher scores in the negative affect of children later [15].

In line with what was stated before, there is a high probability that children in RC would have experienced negative parenting strategies in their early childhood (as the main reason for separation from their parents is parental abuse or neglect), which could have a clear negative impact on their development. In addition, it is also highly probable that they have experienced changes in protective measures, which has a greater impact on those with more reactive and/or dysregulated temperaments, who are more vulnerable to these adverse circumstances. However, once these children are in RC, their care is in the hands of people specifically qualified for the education of children and adolescents (in Spain, these are social educators); this means that it is to be expected that they will use parenting styles adjusted to the needs of this population. Nevertheless, it is worth investigating whether the relationships between fostered children and their care workers can be influenced by the child’s temperament in the same way that bidirectional effects on the parent–child relationship were found.

### 1.3. The Role of Care Workers’ Educational Style 

Care workers (mostly social educators) play a key role in supporting the children’s social and emotional well-being and helping them, as far as possible, in their adaptation and development [18]. Initially, when children are placed in residential care, they usually find it difficult to trust their care workers [19]. Despite this, research shows that with stable and significant contact, it is possible for these young people to build trustful relationships that can help them create other strong and significant bonds [20]. This means that when the RC enhances positive relationships between staff and children, it helps them develop a less insecure attachment style and improves their emotional regulation [21,22]. Research by Costa et al. [19] found that the perception of emotional closeness between residential care workers and fostered children was linked to a positive psychological outcome in adolescents. In Spain, Pérez-García et al. [23] found that, in general, relationships with staff are one of the aspects rated most positively by young people in residential care, while rules and discipline in the care home are viewed more negatively. A recent study by Hernandez et al. [24] that explores the discipline strategies used by residential care workers according to multi-informant reports (i.e., children/adolescents, care workers, and care home managers) showed how frequent punitive strategies that violate children’s rights are used, prompting the authors to stress the need to provide care workers with extra training in the use of more appropriate discipline strategies.

In line with the above, understanding children’s temperament can help care workers develop appropriate expectations for children’s behaviors in certain situations, so that bidirectional effects do not lead to negative educational strategies in care workers, which, in turn, will provoke further negative behaviors in children.

The present study seeks to deepen our understanding of the reciprocal relationships between the caregivers’ discipline styles and the temperament of the youth in RC. Specifically, it aims to find out whether different temperamental characteristics in reactivity and self-regulation are related to and/or can predict the care workers’ discipline styles and the affective relationships they maintain with the children. To this end, this study (a) assessed temperamental reactivity in children placed in RC; (b) evaluated the self-regulation capacity of these young people; (c) examined the discipline styles (inductive, rigid, or permissive) used by care workers and the affection perceived by fostered children; (d) analyzed the relationships between the temperament of youth in RC and the perceived affective relationships with care workers and their discipline styles; and, lastly, (e) analyzed whether temperamental and self-regulation characteristics can predict the care workers’ parenting styles. Based on previous findings, we expect that temperament and self-regulation characteristics will be linked with different discipline styles used by professionals. In addition, it is expected that the temperamental characteristics and lack of regulation of children in care will partly predict the use of different parenting styles by caregivers.

## 2. Materials and Methods

### 2.1. Participants

The participants were all of the children and adolescents residing in residential care homes in the province of Malaga (except for those providing specialist care) during the period between January 2022 and May 2024 who met the following inclusion criteria: (a) aged between 9 and 16 years (we excluded adolescents older than 16 because they are usually engaged in programs designed to prepare them for leaving the measure and the transition to adult life, hence their day-to-day lives tend to differ from their younger peers); (b) had resided in the care home for a minimum of three months (this period was chosen as the minimum required for them to have sufficient experience of how the care home functioned and to form an opinion of their relationship with care workers; (c) did not have severe intellectual or functional impairments that might prevent them from understanding and responding independently to questionnaire items; and (d) had the ability to speak and understand Spanish. A total of 144 children and adolescents met these criteria, leaving a final sample of 60 boys (*M* = 13.27, *SD* = 1.79) and 84 girls (*M* = 13.85, *SD* = 1.74).

### 2.2. Measures

Data were gathered through a questionnaire comprising four instruments designed to assess: (1) temperamental scales and factors within this population; (2) young people’s perceptions regarding the affective relationship with care workers; (3) young people’s perceptions regarding the style of discipline used by care workers; and (4) young people’s self-regulation problems assessed by the caregivers.

1. Early Adolescent Temperament Questionnaire-Revised (EATQ-R) [25]. This instrument is a revision of an instrument developed by Capaldi and Rothbart [26] and it assesses temperament and self-regulation via the adaptation of scales used in studies of children and adults [25]. Originally, this self-report comprised 65 items with 12 subscales; however, for the present study, we used only 6 subscales. The resulting instrument comprised 31 items, rated using a 5-point Likert-type scale (1 = *Never true for me*; 5 = *Always true for me*):
  - Affiliation. The desire for warmth and closeness with others, independent of shyness or extroversion (e.g., I enjoy exchanging hugs with people I like).
  - Fear. Unpleasant affect related to anticipation of distress (e.g., I get frightened riding with a person who likes to speed).
  - Frustration. Negative affect related to interruption of ongoing tasks or goal-blocking (e.g., It really annoys me to wait in long lines).
  - High-Intensity Pleasure. The pleasure derived from activities involving high intensities or novelty (e.g., I would like to live in a big city, even if it wasn’t safe).
  - Perceptual Sensitivity. Detection or perceptual awareness of slight, low-intensity stimulation in the environment (e.g., I am very aware of noises).
  - Pleasure Sensitivity. Pleasure related to activities or stimuli involving low intensities, rates, complexities, novelty, and incongruity (e.g., I enjoy listening to the birds sing).
  - The subscale scores are the average of the items belonging to each subscale. Therefore, scores range from 1 to 5 in all of them. Internal consistency (Cronbach’s alpha) scores of the subscales in the present sample were 0.66 (Affiliation), 0.66 (Fear), 0.75 (Frustration), 0.61 (High-Intensity Pleasure), 0.56 (Perceptual Sensitivity), and 0.82 (Pleasure Sensitivity).
2. Affect Scale [27]. This instrument was used to explore the young person’s perception of the affective relationship with care workers. It comprises 20 items distributed across two factors (10 items each), rated using a 4-point Likert-type scale (1 = *Strongly disagree*; 4 = *Strongly agree*). Scores for each factor therefore range from 10 to 40.
  - Warmth/Communication. Evaluates the perceived warmth and interest expressed by care workers towards the young person (e.g., If you have a problem, can you tell them about it?).
  - Criticism/Rejection explores the perceived degree of criticism that workers show (e.g., Do they ever make you feel like you’re a nuisance?).
  - Internal consistency scores (Cronbach’s alpha) of factors in the present sample were 0.92 (Warmth/Communication) and 0.86 (Criticism/Rejection).
3. Rules and Demands Scale [27]. This instrument was used to explore the young person’s perception of the style of discipline used by care workers. It comprises 30 items distributed across three factors (10 items each), rated using a 4-point Likert-type scale (1 = *Strongly disagree*; 4 = *Strongly agree*). Scores for each factor therefore range from 10 to 40.
  - Inductive style. Refers to the establishment of rules and the reasons why they must be respected (e.g., Do they make it clear what you should or shouldn’t do).
  - Rigid style. Explores the imposition of rules coupled with strong demands that they be respected (e.g., If you disobey, do they punish you?).
  - Permissive style. Refers to the absence of rules and weak demands that they be respected (e.g., If you make a fuss, do you usually end up getting your own way).
  - The internal consistency (Cronbach’s alpha) of scores for the three factors in the present sample was 0.86, 0.75, and 0.69, respectively.
4. Behavior Rating Inventory of Executive Function. Second Edition. Family Version (BRIEF-2) [28]. This instrument was used to explore the executive functioning of this specific population reported by the care workers. The parent form comprises 63 items. This instrument is divided into 12 scales and 4 indexes; however, for the purposes of this study, we only used scores from two scales.
  - Inhibit Problems. Assesses the presence of problems in controlling impulses, regulating behavior appropriately, and restraining it at the appropriate time. (e.g., Is fidgety).
  - Emotional Control Problems. Assesses the presence of problems in regulating or modulating their emotional responses appropriately (e.g., Has explosive, angry outbursts).
  - Each scale contains 8 items rated using a 3-point Likert-type scale (1 = *Never*; 3 = *Frequently*); therefore, scores for both scales range from 8 to 24. Internal consistency scores on these two scales are not available in this study’s population; however, Cronbach’s alpha for these scales was 0.86 (Inhibit Problems) and 0.91 (Emotional Control Problems) for the general population when the Spanish version was validated [28].

### 2.3. Procedures

The research described in this paper is part of a larger collaborative project between the University of Malaga and the child protection service of the regional government in Andalusia (Spain). The project covers all residential care homes for children and adolescents in the province of Malaga, except for those providing specialist care (i.e., for young people with complex or special needs). This corresponds to 22 care homes providing places for around 200 young people.

We first obtained authorization to conduct this study from the Department of Children’s Services of the regional government of Andalusia, as well as from the Research Ethics Committee of the University of Malaga (reference 146-2021-H). Once approval had been granted, we contacted the managers of each of the twenty-two care homes in order to schedule a group meeting with all the children and adolescents living in the home who met the aforementioned inclusion criteria. The object of this meeting was to explain to them the nature and purpose of this study. It was also made clear that participation was entirely voluntary, and that all information would remain anonymous and confidential. There were no rewards/penalties for participation/non-participation. All the young people who met the inclusion criteria agreed to be interviewed, and they signed informed consent prior to any data collection.

With the aim of ensuring that the young person understood each of the questions and that none were skipped, it was decided that the three instruments would be administered by a researcher in the context of an individual interview (rather than as self-reports). All interviews took place at a time and in a room of the young person’s choosing (e.g., their own room or their care worker’s office), ensuring that it would be free from interruptions. Two of the instruments were administered in a first session (Affect Scale and Rules and Demands Scale), another was administered in a second session (EATQ-R), and the last one was administered to the care workers either on paper or through an online survey (BRIEF-2 Family).

### 2.4. Data Analysis

We began by calculating descriptive statistics (mean, standard deviation, and minimum/maximum) for scores on each of the four instruments. Then, we evaluated whether there are significant differences between the scores in the Affect Scale and the Rules and Demands Scale with a pair-sampled T-test. Next, we calculated Spearman’s rho coefficients to examine the association between factor scores on the Affect Scale and the Rules and Demands Scale, and the temperamental subscales (EATQ-R) and self-regulation scales (BRIEF-2). Finally, the statistical technique of multiple linear regression analysis was used to clarify whether some of the variables studied can explain and predict the Warmth/Communication and Criticism/Rejection perceived by the children and the discipline style used by the care workers. All the above analyses were performed using SPSS 25.

## 3. Results

Table 1 and Table 2 present the results obtained with the four instruments we used to evaluate temperamental subscales, children and adolescents’ self-regulation problems assessed by the caregivers, and young people’s perceptions regarding the affective relationship with care workers and the style of discipline used (EATQ-R and BRIEF-2, Affect Scale, and Rules and Demands Scale, respectively). 

To test whether there were significant differences between the scores on the perception of Warmth/Communication or Criticism/Rejection, a paired-sample Student’s T-test was applied. The analysis revealed significant differences between the perception of Warmth/Communication (*M* = 30.27, *SD* = 8.42) and Criticism/Rejection (*M* = 18, *SD* = 6.34), with the perception of Warmth/Communication being higher than Criticism/Rejection (*t*(143) = 10.60, *p* < 0.001).

The same analysis was carried out to determine whether there were significant differences between the scores on the discipline style used by the care workers (Inductive, Rigid, and Permissive). The analysis revealed that children perceive the Inductive style as the most frequently used by care workers (*M* = 30.88, *SD* = 6.83), followed by the Rigid (*M* = 24.16, *SD* = 5.91) and then the Permissive style (*M* = 15.38, *SD* = 4.25). They also showed that there are significant differences between the scores on these scales, with the Inductive style being significantly higher than the Rigid (*t*(143) = 8.57, *p* < 0.001) and Permissive (*t*(143) = 25.13, *p* < 0.001) styles. On the other hand, the score for the rigid style was significantly higher than the permissive style (*t*(143) = 15.15, *p* < 0.001).

### 3.1. Association Between Factor Scores of the Affect Scale and the Rules and Demands Scale with the Temperamental Scales and Factors and Children’s Self-Regulation Problems

Table 3 shows correlations (Spearman’s rho) between factor scores on the affective relationship with care workers and the style of discipline used by them with the temperamental subscales and children and adolescents’ self-regulation problems. It can be seen that Warmth/Communication was significantly and positively associated with Affiliation and Fear, while it was significantly and negatively associated with High-Intensity Pleasure. Conversely, Criticism/Rejection showed a significant positive correlation with High-Intensity Pleasure and Emotional Control Problems. An Inductive Discipline Style was positively associated with the temperamental subscale Fear and negatively correlated with High-Intensity Pleasure, while a Rigid Discipline Style showed the inverse tendency: it was negatively associated with Fear and positively correlated with High-Intensity Pleasure.

### 3.2. Predicting the Affective Relationship with Care Workers from Children and Adolescent’s Temperamental and Self-Regulation Problems Scores 

In the first regression analyses, we examined the prediction of perceived Warmth/Communication from children’s temperamental and self-regulation problems subscales (see Table 4). Three temperament subscales and one self-regulation factor contributed significantly to predicting the perceived Warmth/Communication, and together they accounted for 26% of the total variance: Fear: *F*(1, 142) = 25.40, *p* < 0.001, *R*^2^ = 0.15; Emotional Control Problems: *F* change(2, 141) = 17.26, *p* < 0.001, *R*^2^ change = 0.20; Affiliation: *F* change(3, 140) = 13.68, *p* < 0.001, *R*^2^ change = 0.23; and High Intensity Pleasure: *F* change(4, 139) = 11.90, *p* < 0.001, *R*^2^ change = 0.26. 

A second set of analyses examined the prediction of negative affective relationships with care workers from children’s temperamental and self-regulation problems scales (see Table 4). The temperamental subscale High-Intensity Pleasure and the factor Emotional Control Problems from BRIEF-2 contributed significantly to the prediction of perceived Criticism/Rejection in this analysis: High-Intensity Pleasure: *F*(1, 142) = 19.61, *p* < 0.001, *R*^2^ = 0.12; and Emotional Control Problems: *F* change(2, 141) = 14.69, *p* < 0.001, *R*^2^ change = 0.17. In combination, these factors accounted for 17% of the variance of ratings of perceived Criticism/Rejection.

### 3.3. Predicting the Discipline Style Used by Care Workers from Children and Adolescent’s Temperamental and Self-Regulation Problems Scores

In these analyses, we only included the discipline styles that showed significant correlations with the temperamental and self-regulation problems subscales. In the first regression analyses, we examined the prediction of the use of an inductive discipline style from children’s temperamental and self-regulation problems subscales (see Table 5). One temperament subscale and one self-regulation factor contributed significantly, and together they accounted for 14% of the total variance: Fear: *F*(1, 142) = 15.86, *p* < 0.001, *R*^2^ = 0.10; and Emotional Control Problems: *F* change(2, 141) = 11.10, *p* < 0.001, *R*^2^ change = 0.14. 

A second set of analyses examined the prediction of care workers using a rigid discipline style from children’s temperamental and self-regulation problems scales (see Table 5). Two temperamental subscales (High-Intensity Pleasure and Frustration) and one self-regulation factor from BRIEF-2 (Inhibition Problems) contributed significantly to the prediction of the use of a rigid style: Fear: *F*(1, 142) = 9.63, *p* = 0.002, *R*^2^ = 0.06; Inhibition Problems: *F* change(2, 141) = 9.68, *p* < 0.001, *R*^2^ change = 0.12; and Frustration: *F* change(3, 140) = 8.07, *p* < 0.001, *R*^2^ change = 0.15. In combination, these factors accounted for 15% of the total variance. 

## 4. Discussion

The present study is part of a larger collaborative project with the child protection service of the regional government in Andalusia (Spain). Considering that this is a highly vulnerable population, ethical factors (such as anonymity, willingness to volunteer, informed consent, and respect) were addressed during all phases of this study and we strived to avoid any undesired effects (reliving traumatic situations). From this perspective, this project seeks to find key elements to improve the quality of life, well-being, and adaptation of children in RC, so that professional interventions are consistent with the children’s needs. 

Therefore, this study sought to analyze the relationships between the temperamental characteristics of children in RC (reactivity and self-regulation) and the perceived acceptance/rejection and style of discipline used by care workers, and find out whether the temperamental traits partly explained the characteristics of the educational styles used by the care workers, for example, whether there were child-evoking effects on the parenting styles of care workers due to the temperamental characteristics of children in this context. In general, we found that there was a relationship between the temperamental traits of the children and the parenting styles of the professionals. Regarding the evocative effects of children’s temperament, we observed that some temperamental characteristics of the children partially explained the warmth/rejection shown by care workers and, although to a lesser extent, the discipline styles displayed (inductive, rigid, and permissive). There is no previous research about these specific relationships in the field of RC; however, there is research in the family context that had already identified similar associations between parenting styles and certain temperamental characteristics, as well as bidirectional effects between the two.

The children perceived more warmth/communication than criticism/rejection. These are very positive findings because they show that through contact with caregivers, it is possible to build a good-quality trusting relationship [20], offering them the possibility of building a more secure attachment style, which is also important in terms of their ability to regulate emotions [18]. With respect to the style of discipline used by care workers, our participants perceived this to be mostly inductive. Although a rigid style of discipline was not the predominant perception among our participants, the results highlight the importance of ensuring that care home staff are sufficiently trained in the use of more appropriate (inductive) strategies.

Analyzing the relationships found between children’s temperamental characteristics and the affection they perceived from their care workers, the most significant positive associations were those between Affiliation and Fear with Warmth/Communication, while the negative ones were those between High-Intensity Pleasure with Warmth/Communication and Affiliation and Fear with Criticism/Rejection.

Children’s Affiliation traits (feelings of warmth, closeness, interest, and involvement with others) were found to be related to higher perceptions of care workers’ Warmth/Communication and lower perceptions of Criticism/Rejection. This result was expected and is in line with the findings of other studies. For example, Meuronen et al. [29] also found that high parental Warmth was related to higher adolescent Affiliation. These authors point out that parental Warmth may help adolescents to develop their skills to create close relationships with others; in this sense, in our population, the fact that they perceive their caregivers as caring and communicative may enhance their desire to feel close and open to others, which is very positive in adolescence.

Another interesting and significant relationship is the positive association between Fear (unpleasant affect associated with anticipations of discomfort) and the Affect Scale factors; the greater this temperamental trait is in children, the more Warmth/Communication they perceive from their care workers (and the less Criticism/Rejection). In this case, there is a direct relationship between a measure of a negative temperament affect [30] and the positive educational strategies of care workers. It is possible that this result is due to the particular context in which this study was conducted. The young people in RC have experienced very adverse circumstances with their biological families; many of them have also undergone changes in protection measures and/or changes in RC homes. These are all very unpredictable and unsafe situations that can induce them to feel highly unprotected; therefore, it is possible that the professionals working with them, who are very familiar with their past, tend to be warmer and more communicative and caring with children who show more signs of fear. Lengua and Kovacs [17] and Lengua [31] found, in other contexts, that children’s fear elicited fewer negative parenting behaviors, e.g., higher fear scores predicted increased maternal acceptance and less rejection. According to these authors, signs of fear may lead parents to try to protect or calm their fearful children and/or fearful children may be less difficult to raise, so they enhance more positive parenting strategies.

The highly significant relationship between High-Intensity Pleasure (pleasure related to situations involving high intensities, rates, complexities, novelty, and stimulus incongruity) and perceived affect is also worth noting. Specifically, this temperamental trait has been found to be negatively related to Warmth/Communication and positively related to Criticism/Rejection. Although no previous studies have directly linked High-Intensity Pleasure and parental warmth or affection, a relationship has been found between negative parenting strategies and high scores for this trait, along with externalizing behavioral problems [32]. It seems that, in general, when children are more daring and more novelty- and risk-seeking, caregivers tend to be less warm and more rejecting. Even though this relationship needs further investigation, a possible explanation for these findings could be that care workers are less laid-back or more alert when caring for children characterized by novelty-seeking and this leads to more perceived criticism rather than warmth by the children. It is worth mentioning that other studies have also shown positive associations between surgency (which includes High-Intensity Pleasure) and parental negativity [33], although this could be explained by other characteristics that may be associated with surgency, such as impulsivity or a tendency to rage [34].

A positive relationship was also found between Pleasure Sensitivity and perceived Warmth/Communication. Although no previous studies have specifically highlighted this association, several studies show a relationship between positive emotionality (including Pleasure Sensitivity) and parental warmth [17]. Thus, in the context of RC, it would be expected that caregivers would be more responsive to those children who show more positive emotional traits.

Analyzing the relationships between children’s self-regulation problems and the Affect Scale, the only associations found were with care workers’ Criticism/Rejection. The greater the children’s problems were with behavioral inhibition and emotional control, the greater the perception of criticism and rejection. However, no significant relationships were found between these problems and perceived Warmth/Communication. Other studies with adolescents in the family context did find significant relationships between warmth and affection and better self-regulation [29]. This difference from our findings might be due to the use of different instruments to measure behavioral regulation and affection. For example, in our study, children’s self-regulation problems were assessed through the care workers’ point of view, while in other studies, self-reports were used. On the other hand, several studies use a single-factor measure to assess parental warmth instead of a two-factor scale such as ours. However, the results are consistent regarding less warmth and/or more criticism being associated with greater self-regulation difficulties.

Regarding the relationships found between children’s and adolescents’ temperament and discipline styles displayed by the caregivers, significant relationships were also found with Affiliation, Fear, High-Intensity Pleasure, and Pleasure Sensitivity. Specifically, positive relationships were found between Affiliation, Fear, and Pleasure Sensitivity and the Inductive Discipline Style. In several studies in the family context, positive relationships between appropriate (inductive) discipline styles and positive temperamental traits such as Affiliation and Pleasure Sensitivity were found. On the other hand, the results regarding the temperamental trait Fear are in line with what Lengua [31] found, i.e., a direct relationship with the use of an Inductive style and an inverse relationship with the use of a Rigid style, meaning that it is easier for care workers to use inductive and non-coercive discipline styles with children with higher scores in this temperamental trait. On the other hand, a Rigid style showed weaker positive relationships with High-Intensity Pleasure and Self-regulation Problems than those found with Criticism/Rejection. Again, in line with the studies mentioned above, it seems that care workers tend to use more negative parenting strategies with less-regulated and more novelty-seeking children.

Finally, regarding the evocative effects of children’s temperament on the discipline style displayed by their caregivers, we observed that some temperamental traits of children in RC (namely Fear, Emotional Control Problems, Affiliation and High-Intensity Pleasure) partly explained the Warmth/Communication perceived. On the other hand, High-Intensity Pleasure and Emotional Control Problems explained part of the perceived Criticism/Rejection of care workers. Although the temperamental characteristics of children were correlated to the caregivers’ discipline styles (Inductive, Rigid, and Permissive), they explained less of the variability. Nevertheless, once again, Fear and fewer Emotional Control Problems partly explained the use of an Inductive style, while low levels of Fear and greater problems of Inhibition and Frustration partly explained the use of a Rigid style.

The results of this study are consistent with studies in the family context [10,33,34]. In general, these studies show that more negative temperamental characteristics (such as negative affectivity or worse self-regulation) evoke negative parenting strategies and vice versa. Studies with adolescents have already shown the evocative effects that link adolescents’ negative emotionality with worse parenting strategies (less warmth and worse discipline styles) [34]. It is worth mentioning that in our study, Fear, Emotional Control Problems, Affiliation, and High-Intensity Pleasure explained an important percentage of the variability of the perceived affect, while High-Intensity Pleasure and Emotional Control Problems also explain part of the variability of criticism and rejection. This knowledge is essential for the role of care workers since their main pedagogical tools to avoid problems in adjustment in these children is greater acceptance and less rejection. This means that it would be convenient for care workers to be able to use appropriate educational strategies in all cases, instead of letting themselves be influenced by different temperamental patterns, which can lead to inappropriate educational styles.

Temperamental traits also explained part of the use of Inductive and Rigid strategies. In the family context, recent studies have found a bigger predictive effect between temperament and positive styles than between temperament and negative styles [34]. In our study, the predictive effect was similar for the Inductive and Rigid styles. Once again, the Fear and Self-Regulation scales explained part of the variability of both styles. However, for the rigid style, the temperamental trait Frustration also took part in the explanation. Although these results do not account for most of the variability, it would be interesting for care workers to take them into consideration, since, as we have already pointed out, it is essential to use positive educational strategies with all the children.

In line with the aforementioned, understanding children’s temperaments can help their caregivers develop appropriate expectations for children’s behaviors in certain situations, so that the bidirectional effects do not lead to negative educational strategies in care workers, which in turn evoke more negative behaviors in children.

This study has a number of limitations. The first is the limited number of participants, as the data were gathered from a sample of young people and residential care homes in a specific geographic location. It is very difficult to gain access to this population, as government permissions are difficult to obtain. Increasing the overall sample size and involving children from other regions could allow better analysis of the relationships between the different dimensions of temperament and parenting styles in this specific context.

Another limitation is that all variables were assessed by questionnaires, and these measures have their own limitations due to the effects of informant bias and judgments based on nonobjective criteria. The inclusion of multiple assessment methods, such as behavioral or physiological measures or direct observations, would result in greater reliability of the findings. It would also be interesting if different informants had reported about the study variables (e.g., care workers responding about their own discipline style). Likewise, it would be interesting to make direct observations of the relationship between care workers and foster children to obtain a greater robustness of the associations found. However, in an ethical reflection of this study, the use of direct observation could be controversial due to the inherent vulnerability of these children. Thus, we conclude that the perception of the children themselves provides valuable information that is respectful of the ethical aspects of this study.

The third limitation, and perhaps the most important, is that this study has a cross-sectional design, so we are not able to test the bidirectional effects demonstrated by the other studies mentioned [33,34]. This is one of the most significant limitations, since our study only allows us to identify associations between variables, not causality. This limitation is key to understanding dynamic and complex processes, such as the ones we are studying. In these processes, evolution and changes that take place over time are essential for a better interpretation. It would be interesting to conduct a new longitudinal study that would enable more robust statistical analyses to study the evolution of bidirectional effects between care workers’ discipline styles and temperamental traits in this population. A fourth and very important limitation, which also affects the robustness of the results, is that, in some subscales of the instrument used to assess temperament, moderate levels of reliability, which could affect the associations analyzed, have been obtained. However, the psychometric data obtained did not deviate substantially from those reported for the original version of the scale [25].

Finally, the young people evaluated the care workers of their RC home as a whole, meaning that they were not asked about a particular professional; instead, they answered thinking about how the majority behave. Asking about each specific care worker was not completely possible since in the residential homes, the children are not always assigned a tutor, and there are often many rotations and/or job dropouts. For this reason, we wanted to evaluate the general discipline style used by these professionals.

## 5. Conclusions and Implications

This study contributes to the understanding of the relationship between the temperamental characteristics of children and the educational styles of their caregivers (type of discipline and affect shown) in a very specific context in which no similar studies have been carried out. In general, the results are highly significant as they highlight the relationship between the variables studied and confirm what previous studies with the general population have shown: children’s temperamental characteristics are associated with and partially explain or evoke the educational styles used by their caregivers. In particular, the results encourage further exploration of these relationships to understand more precisely the bidirectional effects found in the reviewed studies and to provide care workers with tools to improve their skills in caring for these children.

In this regard, considering the transactional relationships between parenting and temperament can improve professionals’ actions in caring for children. Therefore, it is essential that care workers’ interventions take temperamental characteristics into account, since the children who are especially vulnerable to the effects of negative parenting (such as lack of affection, rejection, inflexibility, or permissiveness) are precisely the children who evoke this kind of behaviors in caregivers. It would be highly beneficial for care workers to be trained in becoming fully aware of their own conduct and reactions to foster children, in order to identify their educational strategies and adapt them to the temperament of the children (especially for those with greater negative emotionality and poorer regulation).

## Figures and Tables

**Table 1:** Mean (*M*), standard deviation (*SD*), and range of the subscales of EATQ-R and BRIEF-2 (*n* = 144 children/adolescents).

Instrument	Subscales	*M*	Range	*SD*
EATQ-R	Affiliation	3.76	1.40–5.00	0.75
	Perceptual Sensitivity	3.72	1.75–5.25	0.76
	Pleasure Sensitivity	3.09	1.00–5.00	1.17
	Fear	2.87	1.00–4.83	0.90
	Frustration	3.51	1.14–5.00	0.85
	High-Intensity Pleasure	2.92	1.00–5.00	0.76
BRIEF-2	Inhibition Problems	14.84	8.00–24.00	0.75
	Emotional Control Problems	14.72	8.00–24.00	0.76

**Table 2:** Mean (*M*), range, and standard deviation (*SD*) of the factors of the Affect Scale and the Rules and Demands Scale (*n* = 144 children/adolescents).

Instrument	Subscales	*M*	Range	*SD*
Affect Scale	Warmth/Communication	30.27	10–40	8.42
	Criticism/Rejection	18.01	10–37	6.34
Rules and	Inductive style	30.88	14–40	6.83
Demands	Rigid style	24.16	10–39	5.91
Scale	Permissive style	15.38	10–30	4.25

**Table 3:** Correlations (Spearman’s rho) between factor scores on the Affect Scale, the Rules and Demands Scale, and EATQ-R and BRIEF-2.

Variables		Affect Scale		Rules and Demands Scale		
		Warmth/ Communication	Criticism/ Rejection	Inductive Style	Rigid Style	Permissive Style
EATQ-R	Affiliation	0.32 ***	−0.19 *	0.26 **	−0.09	0.02
	Fear	0.39 ***	−0.27 **	0.31 ***	−0.26 **	0.06
	Frustration	0.10	0.06	0.13	0.07	−0.01
	High-Intensity Pleasure	−0.35 ***	0.32 ***	−0.27 *	0.20 *	−0.09
	Perceptual Sensitivity	0.03	0.06	0.01	0.10	−0.08
	Pleasure Sensitivity	0.25 **	−0.15	0.25 **	−0.15	0.18 *
BRIEF-2	Inhibition Problems	−0.09	0.24 **	−0.14	0.21 *	0.02
	Emotional Control Problems	−0.15	0.28 ***	−0.13	0.17 *	0.02

Note. * *p* < 0.05, ** *p* < 0.01, *** *p* < 0.001 (two-tailed).

**Table 4:** Regression model for the effect of temperament and the BRIEF-2 subscales on the factors of the Affect Scale.

Predictors	*F*	*R* ^2^	*B*	*SD*	*p*
*Warmth/Communication*	(4, 139)				
(Constant)	11.90	0.26	27.56	4.97	<0.001
Fear			2.17	0.88	
Emotional Control Problems			−0.13	0.05	
Affiliation			2.28	0.88	
High-Intensity Pleasure			−1.61	0.70	
*Criticism/Rejection*	(2, 141)				
(Constant)	14.69	0.17	5.36	2.65	<0.001
High-Intensity Pleasure			2.00	0.46	
Emotional Control Problems			0.12	0.04	

**Table 5:** Regression model for the effect of temperament and the BRIEF-2 subscales on the factors of the Rules and Demands Scale.

Predictors	*F*	*R* ^2^	*B*	*SD*	*p*
*Inductive Style*	(2, 141)				
(Constant)	11.10	0.14	29.55	2.91	<0.001
Fear			2.62	0.60	
Emotional Control Problems			−0.11	0.44	
*Rigid Style*	(3, 140)				
(Constant)	8.07	0.15	20.54	2.73	<0.001
Fear			−2.31	0.55	
Inhibition Problems			0.09	0.03	
Frustration			1.22	0.59	

## Data Availability

The data that support the findings of this study are available from the corresponding author upon reasonable request.
